# Chemical Characterization and Oxidative Stability of Medium- and Long-Chain Fatty Acid Profiles in Tree-Borne Seed Oils

**DOI:** 10.1155/2018/2178684

**Published:** 2018-04-01

**Authors:** Da-Som Kim, Hoe-Sung Kim, Kyoung-Tae Lee, Dong-Lee Hong, Sung-Rae Cho, Jeong Hoon Pan, Yong Bae Park, Yang-Bong Lee, Jae Kyeom Kim, Eui-Cheol Shin

**Affiliations:** ^1^Department of Food Science, Gyeongnam National University of Science and Technology, Jinju 52725, Republic of Korea; ^2^Southern Forest Resources Research Center, National Institute of Forest Science, Jinju 52817, Republic of Korea; ^3^Department of Food Science and Technology, Pukyong National University of Science and Technology, Busan 48513, Republic of Korea; ^4^School of Human Environmental Sciences, University of Arkansas, Fayetteville, AR 72701, USA

## Abstract

This study was undertaken to evaluate chemical characteristics and oxidative stability of tree-borne seed oils. A total of 15 different fatty acids were identified in six tree-borne seed oils, which included seven types of saturated fatty acids, four types of monounsaturated fatty acids, and four types of polyunsaturated fatty acids. Japanese camphor tree (JCT) had a high content of medium-chain fatty acids (97.94 ± 0.04%), in which fatty acid composition was distinct from those of the other five plant seed oils. Overall, contents of tocopherols, a type of fat-soluble vitamin, ranged between 3.82 ± 0.04 mg/100 g and 101.98 ± 1.34 mg/100 g, respectively. Phytosterol contents ranged from 117.77 ± 1.32 mg/100 g to 479.45 ± 4.27 mg/100 g, respectively. Of all tree-borne seed oils, *β*-sitosterol was the phytosterol at the highest concentration. Contents of unsaponifiables were between 0.13 ± 0.08 and 2.01 ± 0.02, and values of acid, peroxide, and *p*-anisidine were between 0.79 ± 0.01 and 38.94 ± 0.24 mg KOH/g, 3.53 ± 0.21 and 127.67 ± 1.79 meq/kg, and 2.07 ± 0.51 and 9.67 ± 0.25, respectively. Oxidative stability of tree-borne seed oils was assessed through measurement of oxidation-induction periods. These results should serve as a foundation to identify the potential of tree-borne seed oils in industrial application as well as in providing fundamental data.

## 1. Introduction

In modern society, forest plants and their products have been increasingly viewed as useful resources, which have prompted research on applications of plant-based products and utilization of associated organic compounds with diverse physiological activities [1]. Given the potential utility of plant-based products, in particular plant-based oils, it is surprising that relatively few studies have been conducted on oil resources of forest trees, which may be an important source of functional chemical substances. In general, vegetable oils are obtained from grains, and their major components are fatty acids, accounting for over 90% of ester forms. Vegetable fats contain more unsaturated fatty acids than animal fats, and unsaturated fatty acids have important nutritional and functional properties. In particular, many seed oils contain high levels of unsaturated fatty acids. Of them, the n-6 series of linoleic acid (C18 : 2) generate conjugated linoleic acid (CLA), through microbial or chemical reactions, which is an isomer known to have useful functions in the human body [2, 3]. In addition, the n-3 series of alpha-linolenic acid (C18 : 3, n-3), which are particularly representative fatty acids of perilla oil, can be converted to eicosapentaenoic acid (EPA, C20 : 5, n-3) and docosahexaenoic acid (DHA, C22 : 6, n-3) by desaturation and chain elongation in human bodies [4, 5]. These n-3 series of fatty acids are essential to synthesize brain phospholipids and are known to improve brain function, including learning ability [6]. Vegetable fats also contain high levels of phytosterols and vitamin E, thus have antioxidant activity; these constituents confer oxidative stability for food processing, storage, and distribution [7, 8].

Among vegetable oils that are known as sources of fat-soluble components, there have been many studies on seed oils of legumes and nuts [9, 10], but there have been few studies on seed oils derived from tree-borne seeds [11]. Therefore, in this study, we aimed at characterizing chemical characteristics of tree-borne seed oils: mastic-leaf prickly ash (MPA, Zanthoxylum schinifolium Siebold & Zucc), Japanese camphor tree (JCT, *Cinnamomum yabunikkei* H.Ohba), Korean evodia (KOE, *Euodia daniellii* (Benn.) Hemsl.), Japanese lacquer tree (JLT, *Rhus verniciflua* Stokes), Tung tree (TUT, *Vernicia fordii* (Hemsl.) Airy Shaw), and oriental raisin tree (ORT, *Hovenia dulcis* Thunb.). Although these are plants commonly found in the wild, there has been little research on fat-soluble components of their seed oils. In fact, most literature on the use of plant-based products tend to report pharmacological effects of beverage or root components using leaves or fruits of trees, whereas reports on oils tended to focus on simple topics, such as fatty acid composition. As a follow-up study of our recent report about physicochemical properties of tree-borne seed oils [11], here we identified physicochemical properties of oils extracted from 6 trees, by which we evaluated their potentials as oil resources, and provided fundamental data for their use as oil resources.

## 2. Materials and Methods

### 2.1. Materials

The trees from which we obtained tree-borne seed oils for this study were MPA, JCT, KOE, JLT, TUT, and ORT. Samples were harvested and collected by Southern Forest Resources Research Center, Jinju, Republic of Korea, in 2016, and oils were extracted from seed endosperm, for which seed coats and impurities were removed manually. For solvent extraction, samples were mixed with *n*-hexane (10 times sample weight) and stirred at 4°C for 12 h, followed by concentration using a stream of nitrogen (N_2_) gas, resulting in fat-soluble substances, which were used for experiments [12].

### 2.2. Fatty Acid Composition Analysis

To identify fatty acid composition of the extracted tree-borne seed oils, the seed oils were derivatized to methyl ester using boron trifluoride- (BF3-) methanol before analysis [13]. Approximately 0.1 g of tree-borne seed oils were collected in test tubes, to which 0.5 mL heptadecanoic acid (C17 : 0) (1 mg/mL hexane) was added as an internal reference. After adding 2 mL of 0.5 M NaOH methanol to the test tubes, the tube was heated at 110°C for 10 min using a Reacti-Therm III Heating/Stirring Module (Thermo Fisher Scientific Co., Rockford, IL, USA), which was then cooled to room temperature, and heated again at 110°C after adding 4 mL BF3-methanol for 1 h. After cooling, 2 mL hexane was added to the sample mix and vortexed for 1 min, followed by collection of the hexane layer. Solvent was removed from the hexane layer collected through three repetitions of hexane addition and extraction using nitrogen gas, and then the remaining residue was dissolved with 1 mL hexane again, which was used as the sample for fatty acid analysis. Fatty acids of tree-borne seed oils were analyzed by gas chromatography (GC) using Agilent Technologies 6890N (Agilent Technologies, Santa Clara, CA, USA). An SP-2560 capillary column (100 m × 0.25 mm i.d., 0.25 *μ*m film thickness; Agilent Technologies) was used as the column for analysis, and nitrogen (2.7 mL/min) was used as carrier gas. Temperature of the inlet and the detector was 250°C. The sample ratio to the inlet was set to 10 : 1, and rates of hydrogen and air for flame ionization at the detector were set to 40 mL and 450 mL/min, respectively. The initial oven temperature was 130°C for 5 min and gradually increased to 240°C by 4°C/min, and maintained at 240°C for 15 min. Analyzed results were identified by retention time on the basis of 37 fatty acids reference standards (Supelco 37 FAME, Sigma-Aldrich Co., St. Louis, MO, USA).

### 2.3. Vitamin E (Tocopherol) Analysis

To identify tocopherol contents in tree-borne seed oils, the solvent extraction method was used [9]. For pretreatment of samples, 1 g oil was mixed with hexane up to 50 mL and homogenized for 30 s, and this mixture was passed through a 0.45 *μ*m nylon membrane filter (GE Osmonics Lab Store, Minnetonka, MN, USA). A normal-phase HPLC system (Agilent 1260, Agilent Technologies) with a fluorescence detector was used for analysis. The column for separation was LiChrosorb Si-60 column (4 mm × 250 mm, 5 *μ*m particle size; Hibar® Fertigsäule RT, Merck, Darmstadt, Germany) in a normal phase condition. Mobile phase was 0.85% isopropanol/hexane, and flow rate was set as 1 mL/min. Wavelengths of the detector for vitamin E analysis were 290 nm for excitation and 330 nm for emission. For quantification of tocopherol, reference standards for *α*-, *β*-, *γ*-, and *δ*-tocopherol were used. To assess purity of each tocopherol, the reference standards were dissolved in 25 mL ethanol, and then extinction coefficients were measured by a UV-spectrophotometer (DU-62, Beckman Instruments, Inc., Fullerton, CA, USA). Wavelengths for each isomer were 294 (*α*-T), 297 (*β*-T), 298 (*γ*-T), and 298 (*δ*-T) nm. Purity of *α*-, *β*-, *γ*-, and *δ*-tocopherol was 99.07%, 82.47%, 98.71%, and 89.16%, respectively. When judged based on purity of the reference standards, concentrations of stock solutions for *α*-, *β*-, *γ*-, and *δ*-tocopherols were 1.96, 1.65, 3.65, and 1.80 mg/mL, respectively, and the standard solutions were stored at −40°C for further use. For the calibration of each tocopherol, concentrations range for *α*-, *β*-, *γ*-, and *δ*-tocopherols were 0.25–1.96, 0.21–1.65, 0.46–3.65, and 0.23–1.80 mg/mL, respectively.

### 2.4. Contents of Unsaponifiables and Phytosterols

Contents of unsaponifiables in tree-borne seed oils, particularly phytosterols, the representative unsaponifiables, were analyzed by an alkaline saponification method [10]. We mixed 0.5 g of extracted seed oils with 0.5 mL 5*α*-cholestane (1 mg/mL hexanes), an internal reference, followed by addition of 8 mL of 3% (*w*/*v*) pyrogallol-ethanol solution for antioxidative capacity and 1 mL saturated potassium hydroxide (KOH) solution, which was then well mixed, saponified at 80°C for 1 h, and cooled in an ice bucket. For extraction, 10 mL distilled water and 7 mL hexanes were added to the sample mix and then the supernatant corresponding to the hexane layer was separated. Extraction of the hexane layer was repeated three times. The solvent was completely evaporated from the separated hexane layer under nitrogen gas to concentrate the residue. Weight of the completely concentrated residue was measured and compared with input sample amount, by which residue weight was calculated as the ratio of unsaponifiables.

For phytosterol analysis, derivatization was performed by using the trimethylsilyl group. Unsaponifiables were mixed with 0.5 mL of BSTFA (N, O-bis(trimethylsilyl)trifluoroacetamide + trimethylchlorosilane) that contained 1% trimethylsilyl chloride and 1 mL pyridine, and dissolved again, followed by induction of reaction at 80°C for 1 h. After completion of the reaction, samples were slowly cooled at room temperature and filtered with a 0.45 *μ*m filter to remove particles, which were used as samples for phytosterol analysis. Phytosterol was analyzed by gas chromatography, for which HP-5 column (30 m × 0.32 mm i.d., 0.25 *μ*m in film thickness; Agilent Technologies) was used, and helium (2.7 mL/min) was used as the carrier gas. The oven temperature was 260°C at the beginning, increased to 300°C by 3°C/min, and then maintained at 300°C for 15 min. Temperature of the inlet and the detector was 300°C and 320°C, respectively, and the split ratio was 10 : 1. Detection was performed by a flame ionization detector. For identification and measurement of phytosterols, each reference standard, campesterol, stigmasterol, and *β*-sitosterol, was purchased from Sigma-Aldrich Co., and retention time and peak area were used for identification. Phytosterol quantification was performed using 5*α*-cholestane as the internal standard. To accurately quantify the phytosterols in oils, relative response factors (RRFs) to 5*α*-cholestane were determined based on three replicate analyses. The RRFs were determined according to the following equation:

(1) $$\begin{array}{c} \mathrm{RRF}=\frac{PA_{\mathrm{sterol}}}{W_{\mathrm{sterol}}}\times \frac{W_{\mathrm{is}}}{PA_{\mathrm{is}}}, \end{array}$$

where PA_sterol_ is the peak area of the sterol, W_sterol_ is the mass (mg) of the sterol, PA_is_ is the peak area of the internal standard, and W_is_ is the mass (mg) of the internal standard.

On the basis of the RRFs, phytosterol contents were determined using the following equation:

(2) $$\begin{array}{c} \mathrm{Sterols}\;\;\left(\mathrm{mg}/100\mathrm{mg}\;\;\mathrm{lipid}\;\;\mathrm{extract}\right)=\frac{PA_{\mathrm{sterol}}}{PA_{\mathrm{is}}}\times \frac{1}{\mathrm{RRF}}\times \frac{W_{\mathrm{is}}}{W_{\mathrm{sample}}}\times 100, \end{array}$$

where PA_sterol_ is the peak areas of the sterol, PA_is_ is the peak area of the internal standard, W_is_ is the mass (mg) of the internal standard, and W_sample_ is the mass (g) of the oil.

Commercial standard of Δ5-avenasterol was not available, so that right after peak of *β*-sitosterol was considered as Δ5-avenasterol because of its similar molecular weight and close retention time to Δ5-avenasterol and based on the literature [10].

### 2.5. Acid Value, Peroxide Value, and p-Anisidine Value (p-AV)

Acid value of tree-borne seed oils was measured using the American Oil Chemist's Society (AOCS) method [14]. After adding 1 g oil to a 150 mL conical flask, 30 mL of the solution was mixed with ethanol-ether in 1 : 1 (*v*/*v*) ratio to dissolve oils. To this solution, we added (and mixed well) 100 *μ*L of 1% phenolphthalein as an indicator and then the mix was titrated with 0.1 M potassium hydroxide (KOH)/ethanol solution until it became light red. Acid value was finally identified through a mock experiment without sample.

Peroxide value of seed oils were measured using the AOCS method [15]. After collecting 1 g oil in a 250 mL conical flask, oil samples were dissolved with 25 mL of solution mixed with acetic acid-chloroform in 3 : 2 (*v*/*v*) ratio, to which 1 mL saturated potassium iodide (KI) solution was added. The mix was then shaken for 1 min, incubated in a dark condition for 10 min, and mixed well with 75 mL distilled water. Then, 1 mL of 1% starch solution was added, and the solution was titrated with 0.01 N sodium thiosulfate (Na_2_S_2_O_3_) solution until it became transparent. Peroxide value was finally identified through a mock experiment without sample.

To identify amounts of aldehydes and ketones that are secondary oxidation products generated during acidification of seed oils, *p*-AV was utilized. After mixing 100 mg oil with 25 mL isooctane, optical density (OD) was measured at 350 nm. Following reaction of 2.5 mL of this solution with 0.5 mL of 0.25% (*w*/*v*) *p*-anisidine/acetic acid solution for 15 min, OD was measured at the same wavelength. In this way, OD values before and after reaction with anisidine solution were measured to find *p*-AV value [16].

### 2.6. Color Scale and Brown Color Intensity Using Color Difference Meter and Spectrophotometer

Following transfer of 5 g tree-borne seed oils to Petri dishes for color scale analysis, color scale was measured by a color difference meter (CR-300, Konica Minolta Inc., Tokyo, Japan). Each of *L*^∗^ (0/100 darkness/lightness), *a*^∗^ (±, redness/greenness), and *b*^∗^ (±, yellowness/blueness) in the Hunter scale were measured three times, from which mean values were calculated. Values of the standard plate, used as a reference, were *L*^∗^ = 93.59, *a*^∗^ = 2.62, and *b*^∗^ = 1.88. To measure brown color intensity that is related with browning of seed oils, 200 *μ*L seed oils were added to a 96-well plate, and OD was measured at 420 nm using a spectrophotometer (Multiskan Go, Thermo Fisher Scientific, Waltham, MA, USA). A higher OD value was considered as a higher brown color intensity of oil [11].

### 2.7. Oxidation-Induction Periods

To evaluate oxidative stability depending on physicochemical properties of tree-borne seed oils, a rancimat (892 Professional rancimat, Metrohm AG, Herisau, Switzerland) instrument was used. After 3 g of oil was transferred to a rancimat tube, air was injected to the tube with oil at a rate of 20 L/h and a reaction was performed at 120°C in order to induce forced oxidation. Oxidation products such as formic acid, ketones, aldehydes, and carboxylic acids were measured through an oxidation-detecting sensor, based on which oxidation-induction periods were investigated [11].

### 2.8. Statistical Analysis

All experiments were repeated three times, and the results are expressed as mean ± standard deviation. We compared mean values using Tukey's multiple range test in SAS 9.2 (Statistical Analysis System, Version 9.2, SAS Institute Inc., Cary, NC, USA) program. A *P* value less than 0.05 was considered as statistically significant.

## 3. Results and Discussion

### 3.1. Fatty Acid Composition

Fatty acid analysis results of tree-borne seed oils are shown in Table 1. A total of 15 kinds of fatty acids were identified from six tree-borne seed oils, which included seven types of saturated fatty acids, four types of monounsaturated fatty acids, and four types of polyunsaturated fatty acids. For saturated fatty acids, JCT had 97.94 ± 0.04%, corresponding to the highest level, and the remaining five seed oils contained in the range between 6.76 ± 0.02% and 16.24 ± 0.07%. In particular, JCT showed a high medium-chain fatty acid content (>96%) which is interesting given health implications of these fatty acids. Specially, fatty acids of edible oils are long-chain triglycerides composed of 14–20 carbons, which are absorbed by lymph vessels through a fat-specific digestive process, including emulsification and chylomicron formation. Owing to this long digestion process, long-chain triglycerides are relatively less utilized for energy than are carbohydrates or proteins and are likely to be stored in the body, thus likely causing obesity [17]. In contrast, medium-chain triglycerides that have short fatty acids with 8–12 carbons are absorbed by the liver, through the hepatic portal vein, without formation of chylomicron (the general characteristic of fat digestion) and can be digested without bile or lipase. The medium-chain triglycerides are mostly obtained through hydrolysis of coconut oil, butter, and palm oil and are also found in human breast milk, in which they account for approximately 20% of milk. Because medium-chain triglycerides do not need proteins such as fatty acid binding protein, fatty acid transport protein, and fatty acid translocase for their processing, their oxidation rate is faster than that of long-chain fatty acids, and thus, they tend to be used as an energy source rather than being accumulated in the body [17, 18]. According to previous reports, diets with medium-chain fatty acids had a better thermic effect of food than those with long-chain fatty acids [19–21]. Another study also reported that medium-chain fatty acids had a higher thermic effect of food than long-chain fatty acids, reduced appetite, and increased satiety during a 2-week study, resulting in weight loss [22]. In respect to the practicality of cooking with medium-chain fatty acids, their smoke point is low and bubbles are generated when mixed with other vegetable oils or when they are used for frying. To solve these issues, a study on medium- and long-chain triglycerides that contain a certain amount of long-chain triglycerides with essential fatty acids has been ongoing [23].

For monounsaturated fatty acids, the highest level was 53.34 ± 1.21% found in TUT, and its level ranged from 1.82 ± 0.01% in TUT to 37.19 ± 0.02% in MPA. Oleic acid, although not an essential fatty acid, is known to lower cholesterol levels in the body, contributing to prevention of cardiovascular disease, so that it is a nutritionally significant fatty acid [24]. In addition, monounsaturated fatty acids have possessed a higher oxidative stability than polyunsaturated fatty acids, showing a higher convenience and stability in oil processing [25]. Levels of polyunsaturated fatty acids were high in JLT and KOE (62.01 ± 0.14% and 60.39 ± 0.06%). While MPA, ORT, and TUT also had over 40% of polyunsaturated fatty acid contents, their levels in JCT were as low as 0.73 ± 0.01%. Linoleic acid, which is the representative of omega-6 polyunsaturated fatty acids, is mostly found in plant grains, and it lowers blood cholesterols and inhibits arterial thrombus formation. Linoleic acid levels in JLT were as high as 60.41 ± 0.12%. As omega-6 fatty acids are generally unable to be synthesized in humans and mammals, they are classified as essential fatty acids [26].

Omega-3 fatty acids, which are represented by docosahexaenoic acid (DHA) and linolenic acid, have various nutritional functions. ORT was found to have a high linolenic acid content with 35.13 ± 0.05%, and TUT had 28.21 ± 1.20% of DHA (C22 : 6*ω*3). Omega-3 fatty acids that are represented by DHA and linolenic acid have various nutritional functions, which not only serve as a fuel of metabolism but also protect and maintain cells and play important roles in maintenance and apoptosis of heart cells [27]. In addition, linolenic acid helps blood circulation by lowering blood cholesterol levels, affecting prevention or improvement of vascular diseases such as hypertension, arteriosclerosis, angina pectoris, obesity, diabetes, and atopy [27]. The six tree-borne seed oils in this study showed various fatty acid compositions. Some seed oils had a high level of medium-chain fatty acids, others had a high level of monounsaturated fatty acids, and yet others had a high level of polyunsaturated fatty acids. Although polyunsaturated fatty acids are known to function as various biological membranes and are involved in cell metabolism, the human body often fails to synthesize sufficient amounts; therefore, such deficiency needs to be supplemented by an external source (food) [28]. Most seed oils, except JCT oil, contained high levels of unsaturated fatty acids. While contributing to nutritional functions, such high unsaturation levels are vulnerable to acidification. Thus, prevention of acidification for oil storage and processing needs to be studied in order to enable tree-borne seed oils to be used as food and functional food materials.

### 3.2. Tocopherol Contents

Tocopherol contents of tree-borne seed oils are presented in Table 2. A total of three tocopherol isomers (*α*-T, *γ*-T, and *δ*-T) were identified, whereas tocotrienols were undetected. Vitamin E contents in the six tree-borne seed oils ranged from 3.82 ± 0.04 mg/100 g to 101.98 ± 1.34 mg/100 g. KOE oil had the lowest level of tocopherols, whereas oil from ORT had the highest content. Vitamins are organic compounds that support nutrient functions in the body, and tocopherols have antiaging effects due to their antioxidant activity [29]. Tocopherols occur as a viscous, pale-yellow, oily substance in nature and are defined as a derivative group of 2-methyl-6-hydroxy-chroman; in this group, there are four types of tocopherols—*α*, *β*, *γ*, and *δ*—depending on the degree of the methyl group bound to the chroman-ring [9, 29]. For *α*-T, JCT oil had the highest level (10.88 ± 0.43 mg/100 g) among the six tree-borne seed oils. *α*-T has the highest vitamin E activity. *β*-T is present in a trace amount in general vegetable oils but was undetected in all the tree-borne seed oils. *γ*-T is used as antioxidant in major foods and was present in the highest proportion of all tocopherols in the tree-borne seed oils. For *γ*-T, ORT (95.59 ± 1.24 mg/100 g) had the highest level. *δ*-T, which is, like *β*-T, mostly present as a trace element in plants, was found to be at the highest level in JLT (33.92 ± 0.47 mg/100 g), whereas the other oils had only trace amounts of *δ*-T. Tocopherol activity reduces in the order *α*-T > *β*-T > *γ*-T > *δ*-T; tocopherols mainly inhibit oxidation of unsaturated fatty acids, phospholipids, and fat-soluble vitamin A, preventing arteriosclerosis, cataract, and cancer that are caused by lipid peroxide that is generated during lipid acidification. Thus, tocopherols are important vitamins for modern humans [29]. Individual contents in fat-soluble vitamin analysis may vary depending on the extraction method. When Miraliakbari and Shahidi extracted tocopherols from tree nut oils, extraction using chloroform/methanol as solvent showed a significantly higher yield than that of hexane (*P* < 0.05) [12]. It was postulated that such difference in contents could be attributable to a higher solubility in the chloroform/methanol system. While we used hexane in this study, it would vary in tocopherol content depending on the extraction solvent system. As Miraliakbari and Shahidi reported 6.18–19.87 mg/100 g as the range of tocopherol contents in seven tree nut oils [12], it seemed that tocopherol contents of tree-borne seed oils, except that of KOE, should not be less than those of tree nut oils that are used in food.

### 3.3. Phytosterol Contents

Phytosterol contents within tree-borne seed oils are listed in Table 3. Phytosterol contents ranged from 117.77 ± 1.32 mg/100 g in TUT to 479.45 ± 4.27 mg/100 g in JLT. Of phytosterols, campesterol was found at the highest level (59.43 ± 0.31 mg/100 g) in MPA, and JLT had the highest levels of stigmasterol (20.20 ± 0.17 mg/100 g), *β*-sitosterol (388.84 ± 3.19 mg/100 g), and Δ5-avenasterol (34.54 ± 0.74 mg/100 g). According to a literature [30], comparisons of phytosterols extracted from coriander and seed oils of caraway, anise, nutmeg, and white mustard showed that their phytosterol contents ranged between 283.64 and 859.79 mg/100 oil, based on which it was considered that phytosterol contents in tree-borne seed oils except JLT oil were lower than those reported in the literature. Phytosterols refer to sterols originated from plants, and over 250 phytosterols have been reported to date. A clinical trial confirmed that phytosterols lowered cholesterol [31]. As phytosterols are not synthesized in the human body, they need to be provided through diet. The human body has an extremely low level of phytosterol in the blood, which is because of a low intestinal absorption rate of phytosterols [31].

### 3.4. Unsaponifiables

Proportions of unsaponifiables within tree-borne seed oils are presented in Table 4. ORT and JLT had the highest level of unsaponifiables in tree-borne seed oils, which seemed to be proportional to the high tocopherol level (101.98 ± 1.34 mg/100 g oil; Table 2) and a high phytosterol content (479.45 ± 4.27 mg/100 g oil; Table 3) of JLT. For industrial application of unsaponifiables (fat-soluble vitamins, fat-soluble pigments, and phytosterols), the study results on total oil contents of seeds will be informative.

### 3.5. AV, POV, and p-AV

Contents of AV, POV, and *p*-AV in tree-borne seed oils are presented in Table 5. Acid value indicates acidity when fatty acids of oils bound to glycerol were separated to free fatty acids, by the change in quality; a high acid value means a high proportion of free fatty acids or a low quality of oils. Of the oil resources, MPA had the highest acid value with (38.94 ± 0.24), whereas TUT had the lowest value (0.79 ± 0.01). Additional research is needed to determine if the high acid value of MPA was due to acidification or due to preexisting free fatty acids. For POV, JCT oil showed the lowest value (3.53 ± 0.21 meq/kg) among the tree-borne seed oils, whereas MPA oil showed a relatively high value (127.67 ± 1.79 meq/kg). Although POV is a basic method to measure primary oxidation products, it is difficult to use in cases of high temperature or increasing acidification. POV is obtained by measuring lipid hydroperoxide, the primary oxidation product, which is used for detection of acidification in oils or for the measurement of the induction period. At levels of 60 meq/kg or lower (the reference value for POV content of vegetable oils for frying), it is possible that acidification by generation of primary oxidation products (e.g., in tree-borne seed oils except MPA oil) might not have been completed yet [32].

Oil acidification leads to elevation of aldehyde levels, and *p*-anisidine forms yellow complexes by reacting with aldehydes in the presence of acetic acid, so that the degree of acidification is assessed through measurement of such complexes [33]: 2-alkenal or 2,4-alkadienal aldehydes. In general, *p*-AV is a measure for the level of secondary oxidation products among primary and secondary metabolites [34]. In this study, *p*-AV ranged from 2.07 ± 0.51 to 9.67 ± 0.25. Except MPA oil, all tree-borne seed oils had stable values from 2.07 ± 0.51 to 4.18 ± 0.50. In a study of Miraliakbari and Shahidi, oxidative stability of seven tree nut oils were measured, in which *p*-AV ranges of tree nut oils after being freshly collected and after 12 days of storage were between 0.24 and 0.55 and from 9.36 to 48.83, respectively [12]. Considering *p*-AV of tree-borne seed oils, it is likely that aldehydes were formed in all the oils to some degree; thus, it seems to be necessary to block oxygen or maintain low temperature during oil extraction in order to prevent oxidation in future.

### 3.6. Brown Color Intensity and Color Scale

Measured brown color intensities of tree-borne seed oils using as an indicator of oil oxidation are shown in Table 6. To measure browning intensity, we used the absorption of wavelength at 420 nm that specifically absorbs brown colors. Highest browning intensities were observed in JCT and ORT oils (2.84 ± 0.23 and 2.29 ± 0.06), whereas TUT oil had the lowest browning intensity (0.20 ± 0.01). Color scales identified at 420 nm not only included brown color intensity from browning but also reflected changes by each oil's own brown pigment. Further, color scales of oils of tree-borne seed were measured by a color difference meter, and the results are shown in Table 6. Although there was no significant difference in *L*^∗^ values that reflected brightness in the colorimeter, TUT oil had the highest color scale (41.03 ± 0.02). Overall, *a*^∗^ values of color scale had negative values, indicating greenness. ORT oil showed a relatively high greenness (−4.25 ± 0.01). As for the *b*^∗^ values in the color scale, a higher positive value indicates an increase in yellowness, and ORT oil (10.47 ± 0.01) had a relatively high yellowness. Such high yellowness was consistent with browning intensity (2.29 ± 0.06). In addition, TUT oil also was consistent in terms of the lowest browning intensity (0.20 ± 0.01) and the lowest yellowness (0.04 ± 0.01).

### 3.7. Oxidative Stability

**Figure 1:**
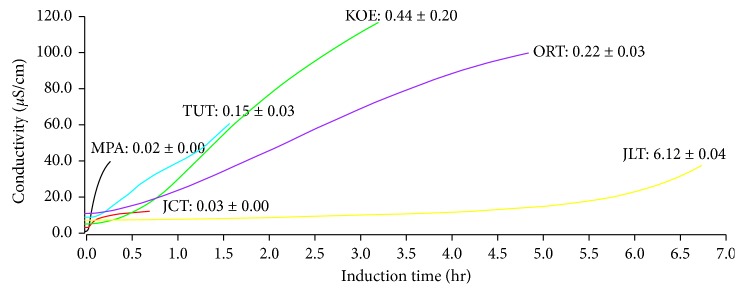
Measurement of induction times of tree-borne oils by the rancimat method.

Oxidative stability of tree-borne seed oils was measured by the rancimat test, and the results are presented in Table 7 and Figure 1. Lipids can produce oxidative compounds such as volatile organic acids (mainly formic acid and acetic acid) during oxidation processes. Oxidation-induction period refers to the time when oxygen absorption rate reached the maximum immediately before acidification. Volatile dicarboxylic acids are generated from acidification of tree-borne seed oils, and they produce secondary oxidative compounds including alcohols, aldehydes, ketones, and low molecular weight products causing electrical conductivity to increase within the rancimat system, by which the oxidation-induction period was assessed. The oxidation-induction period is known to be an indicator of antioxidative activity and oxidative stability of oils [35]. In general, a longer oxidation-induction period means a higher oxidative stability in oils. Of the six types of tree-borne seed oils in this study, JLT oil had the longest oxidation-induction period of 6.12 ± 0.04 h, whereas the shortest oxidation-induction period was observed in MPA oil (0.02 ± 0.01 h). Fatty acid profiles are an important parameter for oxidative stability in oils, but this study shows somewhat different patterns for the induction time compared to the general principle of rancimat method. JLT oil represented a high content of polyunsaturated fatty acids (68%) as well as excellent oxidative stability; in contrast, MPA oil had 49% of polyunsaturated fatty acid yet showed relatively low oxidative stability, these inconsistent trends indicate that induction time cannot be simply explained by a degree of polyunsaturated fatty acid compositions. In fact, the induction times for MPA (0.02 hr) and JCT (0.03 hr) were too short to produce oxidative compounds from the fatty acid decomposition (Figure 1). These induction times can be caused by other naturally occurring volatile compounds in oils prior to oxidation. Chemical parameters of MPA are consistent with these patterns; high AV (38.94 ± 0.24 mg KOH/g), POV (127.67 ± 1.79 meq/kg), and *p*-AV (9.67 ± 0.25) values. Moreover, MPA oil had lower tocopherol content (6.01 ± 0.06 mg/100 g) than others. Therefore, the short oxidation-induction period in MPA oil seemed reasonable. Hidalgo and others demonstrated that the addition of phospholipid to edible oils can increase their induction times [35]. The effect of phospholipid was similar to that of BHT (a synthetic antioxidant) when added [35]. For the comparison between low unsaturated fatty acids and high unsaturated fatty acid, edible oils should be refined because crude oils can contain free fatty acids, phospholipid, and various fat-soluble components which can affect the measurement of induction time. One study reported the various oxidation-induction period of olive oil from 65 to 140 h at low temperatures, demonstrating that edible oil with similar fatty acid profiles may represent varying induction times [36]. The report showed a large difference in the induction time (65–140 h) even though identical oil sample was used; hence, subtle differences in nutrients could result in a broad range of oxidation-induction period lengths. Taken together, it seems that the range of oxidation-induction periods in the six types of tree-borne seed oils may be due to both innate differences in oxidative stability and a reduced quality and stability caused by changes during extraction.

## 4. Conclusion

This work was to identify potential of oil resources extracted from trees and fat-soluble nutrients, and their physicochemical features were investigated. As for nutrients, all the tree-borne seed oils except JCT oil had high levels of unsaturated fat (>80%) and the tree-borne seed oils had variable contents of tocopherol, fat-soluble vitamins, and phytosterols (plant sterols). For physicochemical features, acid values and POVs for the primary oxidation product were stable in all seed oils except MPA oil, and *p*-AV, the indicator for secondary oxidation products, was also identified. For browning intensity and color scale, it was difficult to conclude freshness because the values from these tests also contained oil's own color scale; however, these tests provided fundamental information about color scale. As for the oxidation-induction period, the indicator of oxidative stability, results varied depending on the level of unsaturated fatty acids and *α*-T, fat-soluble antioxidant. This study demonstrated that the chain length and rate of unsaturation in fatty acids can be a parameter for the use of edible oils as a nutritional supplement, the content of each tocopherol acting as antioxidant agent, and the phytosterol in tree-borne seed oils as cholesterol-lowering agent in our body. Physicochemical results in this study will be useful parameters for the refining process of tree-borne seed oils in industrial applications.

## Figures and Tables

**Table 1:** Fatty acid profiles in tree-borne seed oils.

Fatty acid	Composition (%)					
	JCT	KOE	JLT	MPA	ORT	TUT
Capric acid (C10 : 0)	30.22 ± 0.03	nd	nd^∗^	nd	nd	nd
Lauric acid (C12 : 0)	65.83 ± 0.03	nd	nd	nd	nd	nd
Myristic acid (C14 : 0)	1.02 ± 0.01	nd	nd	nd	nd	nd
Palmitic acid (C16 : 0)	0.38 ± 0.01	9.80 ± 0.01	13.82 ± 0.06	11.26 ± 0.03	6.75 ± 0.01	3.84 ± 0.01
Stearic acid (C18 : 0)	nd	2.61 ± 0.01	3.52 ± 0.01	1.85 ± 0.01	3.98 ± 0.01	2.89 ± 0.01
Arachidic acid (C20 : 0)	nd	nd	nd	nd	0.85 ± 0.01	nd
Behenic acid (C22 : 0)	nd	nd	nd	nd	0.43 ± 0.01	nd
ΣSaturated fatty acids	97.94 ± 0.04^a^	12.35 ± 0.02^c^	16.24 ± 0.07^b^	13.24 ± 0.05^c^	12.14 ± 0.02^c^	6.76 ± 0.02^d^
Palmitoleic acid (C16 : 1*ω*7)	nd	5.04 ± 0.01	0.72 ± 0.01	2.38 ± 0.01	0.53 ± 0.01	nd
Oleic acid (C18 : 1*ω*9)	1.82 ± 0.01	21.40 ± 0.09	20.54 ± 0.04	34.01 ± 0.01	31.38 ± 0.04	10.33 ± 0.05
Gondoic acid (C20 : 1*ω*9)	nd	nd	nd	0.50 ± 0.01	nd	nd
Nervonic acid (C24 : 1*ω*9)	nd	nd	nd	nd	nd	42.78 ± 0.76
ΣMonounsaturated fatty acids	1.82 ± 0.01^e^	27.17 ± 0.09^c^	21.08 ± 0.05^d^	37.19 ± 0.02^b^	32.10 ± 0.05^bc^	53.34 ± 1.21^a^
Linoleic acid (C18 : 2*ω*6)	0.73 ± 0.01	38.96 ± 0.04	60.41 ± 0.12	27.84 ± 0.01	18.65 ± 0.01	11.96 ± 0.01
Eicosadienoic acid (C20 : 2)	nd	nd	nd	0.40 ± 0.01	nd	nd
Linolenic acid (C18 : 3*ω*3)	nd	21.95 ± 0.04	0.99 ± 0.01	21.75 ± 0.02	35.13 ± 0.08	nd
Docosahexaenoic acid (C22 : 6*ω*3)	nd	nd	nd	nd	nd	28.21 ± 1.20
ΣPolyunsaturated fatty acids	0.73 ± 0.01^f^	60.39 ± 0.06^b^	62.01 ± 0.14^a^	49.58 ± 0.03^d^	55.01 ± 0.09^c^	40.03 ± 1.32^e^
Oil content (g/100 g)	20.17 ± 1.24^c^	33.59 ± 2.01^a^	24.60 ± 1.73^c^	9.40 ± 0.72^d^	29.6 ± 1.96^b^	4.6 ± 0.28^e^

^∗^Means with different letters (a–f) within a row are significantly different by Tukey's multiple test (*P* < 0.05); nd = not detected.

**Table 2:** Tocopherol (T) composition in tree-borne seed oils.

Sample	Tocopherols (mg/100 g oil)			Total-T
	*α*-T	*γ*-T	*δ*-T	
MPA	6.01 ± 0.06^b^^∗^	nd	nd	6.01 ± 0.06^e^
JCT	10.88 ± 0.43^a^	11.13 ± 0.70^d^	4.78 ± 0.60^c^	26.79 ± 1.72^d^
KOE	nd	3.66 ± 0.05^e^	0.16 ± 0.01^e^	3.82 ± 0.04^f^
JLT	nd	42.47 ± 1.28^b^	33.92 ± 0.47^a^	76.40 ± 1.73^b^
TUT	2.81 ± 0.10^c^	35.52 ± 0.21^c^	2.45 ± 0.04^d^	40.78 ± 0.11^c^
ORT	nd	95.59 ± 1.24^a^	6.39 ± 0.16^b^	101.98 ± 1.34^a^

^∗^Means with different letters (a–f) within a column are significantly different by Tukey's multiple test (*P* < 0.05); nd = not detected.

**Table 3:** Phytosterol contents in tree-borne seed oils.

Sample	Phytosterols (mg/100 g oils)				
	Campesterol	Stigmasterol	*β*-Sitosterol	Δ5-Avenasterol	Total
MPA	59.43 ± 0.31^a^^∗^	5.84 ± 0.10^c^	99.76 ± 0.88^d^	12.25 ± 0.17^c^	177.28 ± 1.29^c^
JCT	12.67 ± 0.19^e^	2.82 ± 0.14^d^	114.98 ± 2.30^c^	10.85 ± 0.58^d^	141.32 ± 3.21^d^
KOE	19.17 ± 0.25^d^	3.14 ± 0.05^d^	90.43 ± 1.12^e^	12.19 ± 0.13^c^	124.93 ± 1.55^e^
JLT	35.87 ± 0.43^b^	20.20 ± 0.17^a^	388.84 ± 3.19^a^	34.54 ± 0.74^a^	479.45 ± 4.27^a^
TUT	5.16 ± 0.06^f^	13.26 ± 0.11^b^	90.73 ± 1.04^e^	8.62 ± 0.14^e^	117.77 ± 1.32^f^
ORT	28.42 ± 1.09^c^	19.31 ± 0.84^a^	161.18 ± 6.59^b^	18.15 ± 0.62^b^	227.06 ± 9.13^b^

^∗^Means with different letters (a–f) within a column are significantly different by Tukey's multiple test (*P* < 0.05).

**Table 4:** Unsaponifiables in tree-borne seed oils.

Sample	Unsaponifiables (%)
MPA	0.39 ± 0.04^d^^∗^
JCT	0.52 ± 0.08^c^
KOE	0.28 ± 0.18^e^
JLT	0.84 ± 0.05^b^
TUT	0.13 ± 0.08^e^
ORT	2.01 ± 0.02^a^

^∗^Means with different letters (a–e) within a column are significantly different by Tukey's multiple test (*P* < 0.05).

**Table 5:** Measurements of acid value (AV), peroxide value (POV), and *p*-anisidine value (*p*-AV) in tree-borne seed oils.

Sample	Acid value (mg KOH/g)	Peroxide value (meq/kg)	*p-*Anisidine value
MPA	38.94 ± 0.24^a^^∗^	127.67 ± 1.79^a^	9.67 ± 0.25^a^
JCT	34.92 ± 1.94^b^	3.53 ± 0.21^e^	2.48 ± 0.48^d^
KOE	9.87 ± 0.43^d^	12.98 ± 0.17^c^	2.52 ± 0.40^d^
JLT	11.32 ± 0.14^c^	8.21 ± 0.18^d^	2.07 ± 0.51^e^
TUT	0.79 ± 0.01^e^	40.86 ± 0.70^b^	4.18 ± 0.50^b^
ORT	9.62 ± 0.48^d^	14.93 ± 0.50^c^	2.84 ± 0.06^c^

^∗^Means with different letters (a–e) within a column are significantly different by Tukey's multiple test (*P* < 0.05).

**Table 6:** Measurements of browning intensity and color scale in tree-borne seed oils.

Sample	Browning	*L* ^∗^	*a* ^∗^	*b* ^∗^
MPA	0.85 ± 0.09^d#^	39.04 ± 0.10^c^	−1.14 ± 0.03^a^	1.30 ± 0.02^d^
JCT	2.84 ± 0.23^a^	37.86 ± 0.01^d^	−1.26 ± 0.03^b^	5.91 ± 0.02^b^
KOE	0.39 ± 0.01^e^	40.57 ± 0.03^b^	−1.39 ± 0.05^b^	1.26 ± 0.03^d^
JLT	1.61 ± 0.19^c^	35.19 ± 0.02^e^	−2.22 ± 0.06^c^	4.45 ± 0.04^c^
TUT	0.20 ± 0.01^f^	41.03 ± 0.02^a^	−1.08 ± 0.02^a^	0.04 ± 0.01^e^
ORT	2.29 ± 0.06^b^	39.53 ± 0.01^c^	−4.25 ± 0.01^d^	10.47 ± 0.01^a^

^#^Means with different letters (a–f) within a column are significantly different by Tukey's multiple test (*P* < 0.05).

**Table 7:** Induction times of tree-borne seed oils.

Sample	Induction time (h)
MPA	0.02 ± 0.00^e^^∗^
JCT	0.03 ± 0.00^e^
KOE	0.44 ± 0.20^b^
JLT	6.12 ± 0.04^a^
TUT	0.15 ± 0.03^d^
ORT	0.22 ± 0.03^c^

^∗^Means with different letters (a–e) within a column are significantly different by Tukey's multiple test (*P* < 0.05).

## References

[1] Park Y. K., Roh H. J., Jeon J. H., Kim H. H. (2010). Analyzing the type and priority order of forest functions for private forests. *Journal of Agricultural & Life Science*.

[2] Ha Y. L., Pariza M. W. (1991). Naturally-occurring novel anticarcinogens: conjugated linoleic acid (CLA). *Journal of the Korean Society of Food Science and Nutrition*.

[3] Hur S. J., Lee J. I., Ha Y. L., Park G. B., Joo S. T. (2002). Biological activities of conjugated linoleic acid (CLA) and animal products. *Journal of the Korean Society of Animal Sciences and Technology*.

[4] Dyerberg J., Bang H. O., Stoffersen E., Moncada S., Vane J. R. (1978). Eicosapentaenoic acid and prevention of thrombosis and atherosclerosis?. *The Lancet*.

[5] Hirai A., Hamazaki A., Jajili J. (1980). Eicosapentaenoic acid platelet function in Japanese. *The Lancet*.

[6] Neuringer M., Connor W. E. (1986). n-3 fatty acids in the brain and retina: evidence for their essentiality. *Nutrition Reviews*.

[7] Gurr M. I., Harwood J. L., Frayn K. N. (2002). *Lipid Biochemistry*.

[8] Lee I. B., Choi K. J., Yu K. K., Chang K. W. (1992). Tocopherols and fatty acids in plant seeds from Korea. *Journal of the Korean Chemical Society*.

[9] Shin E. C., Huang Y. Z., Pegg R. B., Phillips R. D., Eitenmiller R. R. (2009). Commercial runner peanut cultivars in the United States: tocopherol composition. *Journal of Agricultural and Food Chemistry*.

[10] Shin E. C., Pegg R. B., Phillips R. D., Eitenmiller R. R. (2010). Commercial peanut (*Arachis hypogaea* L.) cultivars in the United States: phytosterol composition. *Journal of Agricultural and Food Chemistry*.

[11] Kim M. S., Park J. H., Lim H. J. (2017). Nutritional components and physicochemical properties of lipids extracted from forest resources. *Journal of the Korean Society of Food Science and Nutrition*.

[12] Miraliakbari H., Shahidi F. (2008). Oxidative stability of tree nut oils. *Journal of Agricultural and Food Chemistry*.

[13] Prato E., Biandolino F. (2012). Total lipid content and fatty acid composition of commercially important fish species from the Mediterranean, Mar Grande Sea. *Food Chemistry*.

[14] AOCS (2017). Acid value of fats and oils. *Official Methods and Recommended Practices of the AOCS*.

[15] AOCS (2017). Peroxide value, acetic acid, isooctane method. *Official Methods and Recommended Practices of the AOCS*.

[16] AOCS (2017). p-anisidine value. *Official Methods and Recommended Practices of the AOCS*.

[17] Bach A., Debry G., Metais P. (1977). Hepatic metabolism of medium-chain triglyceride. *Bibliotheca Nutritio Et Dieta*.

[18] Bach A. C., Babayan V. K. (1982). Medium-chain triglycerides: an update. *American Journal of Clinical Nutrition*.

[19] Seaton T. B., Welle S. L., Warenko M. K. (1986). Thermic effect of medium-chain and long-chain triglycerides in man. *American Journal of Clinical Nutrition*.

[20] Hill J. O., Peters J. C., Yang D. (1989). Thermogenesis in humans during overfeeding with medium-chain triglycerides. *Metabolism*.

[21] Dulloo A. G., Fathi M., Mensi N., Cirardier L. (1996). Twenty-four-hour energy expenditure and urinary catechol amines of humans consuming low-to-moderate amounts of medium-chain triglycerides: a dose-response study in human respiratory chamber. *European Journal of Clinical Nutrition*.

[22] Wymelbeke V. V., Louis-Sylvestre J., Fantino M. (2001). Substrate oxidation and control of food intake in men after a fat-substitute meal compared with meals supplemented with an isoenergetic load of carbohydrate, long-chain triacylglycerols, or medium-chain triacylglycerols. *American Journal of Clinical Nutrition*.

[23] Koh S. P., Arifin N., Lai O. M., Yusoff M. S. A., Long K., Tan C. P. (2009). Medium-chain fatty acids–nutritional function and application to cooking oil. *Journal of the Science of Food and Agriculture*.

[24] Noh S., Yoon S. H. (2012). Stereospecific positional distribution of fatty acids of Camellia (*Camellia japonica* L.) seed oil. *Journal of Food Science*.

[25] O’Keefe S. F., Wiley V. A., Knauft D. A. (1993). Comparison of oxidative stability of high-and normal-oleic peanut oils. *Journal of the American Oil Chemists’ Society*.

[26] Watkins S. M., German J. B., Akoh C. C., Min D. B. (2002). Unsaturated fatty acids. *Food Lipids*.

[27] Nelson G. J., Chow C. K. (1992). Dietary fatty acids and lipid metabolism. *Fatty acids in Foods and Their Health Implications*.

[28] Timilsena Y. P., Vongsvivut J., Adhikari R., Adhikari B. (2017). Physicochemical and thermal characteristics of Australian chia seed oil. *Food Chemistry*.

[29] Eitenmiller R. R., Lee J. (2004). *Vitamin E: Food Chemistry, Composition, and Analysis*.

[30] Kozłowska M., Gruczyńska E., Ścibisz I., Rudzińska M. (2016). Fatty acids and sterols composition, and antioxidant activity of oils extracted from plant seeds. *Food Chemistry*.

[31] Moreau R. A., Whitaker B. D., Hicks K. B. (2002). Phytosterols, phytostanols, and their conjugates in foods: structural diversity, quantitative analysis, and health-promoting uses. *Progress in Lipid Research*.

[32] Lee K. S., Kim G. H., Kim H. H. (2013). Physicochemical properties of frying ginseng and oils derived from deep-frying ginseng. *Journal of the Korean Society of Food Science and Nutrition*.

[33] Akoh C. C., Min D. B. (2002). *Food Lipids-Chemistry, Nutrition, and Biotechnology*.

[34] Naz S., Sheikh H., Siddiqi R., Sayeed S. A. (2004). Oxidative stability of olive, corn, and soybean oil under different conditions. *Food Chemistry*.

[35] Hidalgo F. J., Leon M. M., Zamora R. (2006). Antioxidative activity of amino phospholipids and phospholipid/amino acid mixtures in edible oils as determined by the rancimat method. *Journal of Agricultural and Food Chemistry*.

[36] Mancebo-Campos V., Salvador M. D., Fregapane G. (2007). Comparative study of virgin olive oil behavior under rancimat accelerated oxidation conditions and long-term room temperature storage. *Journal of Agricultural and Food Chemistry*.

